# Glymphatic and meningeal lymphatic dysfunction in Alzheimer's disease: Mechanisms and therapeutic perspectives

**DOI:** 10.1002/alz.70709

**Published:** 2025-10-28

**Authors:** Jiangwei Ding, Chengbin Zhao, Xiaoyan Hao, Hongliang Jiao

**Affiliations:** ^1^ Department of Neurosurgery The First Affiliated Hospital of Zhengzhou University Zhengzhou City Henan China; ^2^ Department of Neurology The First Affiliated Hospital of Zhengzhou University Zhengzhou City Henan China

**Keywords:** β‐amyloid clearance, Alzheimer's disease, glymphatic system, lymphatic–venous anastomosis, neuroinflammation, sleep

## Abstract

Alzheimer's disease (AD) is a neurodegenerative disorder characterized by amyloid‐beta (Aβ) deposition and tau pathology. Although disease‐modifying therapies, such as anti‐Aβ monoclonal antibodies, have been approved, their clinical efficacy remains modest and accompanied by substantial safety concerns. The glymphatic system, which is a brain‐wide waste clearance network mediated by cerebrospinal fluid‐interstitial fluid exchange, is critical in AD pathogenesis. Glymphatic dysfunction promotes Aβ and tau accumulation, neuroinflammation, and vascular impairment, forming a vicious cycle that drives neurodegeneration. This review elucidates the anatomical and physiological basis of the glymphatic system, its role in AD progression, and novel therapeutic strategies targeting glymphatic enhancement. Emerging interventions, including aquaporin‐4 (AQP4) modulation, meningeal lymphatic regeneration, cervical deep lymphaticovenous anastomosis, and nonpharmacological approaches, are also discussed for their potential to shift AD therapeutics from symptom management to disease modification.

**Highlights:**

- Glymphatic dysfunction drives Alzheimer's disease (AD) pathogenesis.
- Meningeal lymphatic decline with aging.
- Cervical lymphaticovenous anastomosis (LVA) as novel new intervention method for patients with AD
- The apolipoprotein E (APOE) ε4 allele disrupts meningeal lymphatic function, increasing AD risk via amyloid‐β (Aβ) clearance deficits.
- Diffusion tensor imaging along the perivascular space (DTI‐ALPS) and dynamic magnetic resonance imaging (MRI) enable early detection of glymphatic dysfunction, guiding pre‐symptomatic AD interventions.
- Sleep and arterial pulsatility critically regulate glymphatic efficiency, offering non‐pharmacological therapeutic avenues.

## 1 INTRODUCTION

Alzheimer's disease (AD) is a progressive neurodegenerative disorder characterized by progressive memory impairment and cognitive decline, severely impairing patients' quality of life and shortening life expectancy.^1^ AD has become a critical public health challenge and imposes substantial economic burdens on families and society,^1^, ^2^ with its global prevalence projected to exceed 100 million by 2050.^3^ According to the China Alzheimer Report 2024, there are currently nearly 17 million dementia patients in China, accounting for approximately 30% of the global total. AD is the most common type of dementia among older people.

The classical pathological model of AD centers on extracellular amyloid‐β (Aβ) deposition and intracellular neurofibrillary tangles composed of hyperphosphorylated tau.^4^, ^5^ However, this model does not fully explain the failure of clearance mechanisms. Although the anti‐Aβ monoclonal antibody lecanemab has been approved by the United States Food and Drug Administration (FDA) for mild AD, its efficacy and safety remain controversial.^6^, ^7^, ^8^ Reported adverse events include amyloid‐related imaging abnormalities (ARIA) of the hemorrhagic (ARIA‐H) and edematous (ARIA‐E) types, infusion‐related reactions, and even death.^6^, ^9^

The dynamics of solute exchange between the cerebrospinal fluid (CSF) and brain parenchyma have long been debated. Traditional models have postulated that the solute exchange primarily occurs through diffusion driven by solute concentration gradients,^10^ which was confirmed in a recent study by Decker et al.^10^ However, in 2012, Maiken and colleagues proposed the glymphatic system, a unique waste clearance system in the brain whose function depends on aquaporin‐4 (AQP4) located in astrocytic endfeet.^11^ In 2015, Louveau et al.^12^ discovered functional lymphatic vessels within the dural sinuses of mice. The cells in these vessels exhibited molecular characteristics of lymphatic endothelial cells, demonstrated the capacity to transport fluid and immune cells from the CSF, and were anatomically connected to the deep cervical lymph nodes (dcLNs). This groundbreaking discovery provided evidence linking the glymphatic system to the peripheral lymphatic circulation. In the same year, Aspelund et al.^13^ also discovered that meningeal lymphatic vessels can absorb the CSF from the adjacent subarachnoid space and brain interstitial fluid (ISF) and transport it to the dcLNs via the foramina at the base of the skull. Similarly, Albayram et al.^14^ identified the presence of meningeal lymphatic vessels in the human brain. They further discovered that these lymphatic pathways, aligned with cranial nerves and vascular structures, are directly connected to cervical lymph nodes. They also demonstrated reduced lymphatic outflow in the aging brain by using high‐resolution magnetic resonance imaging (MRI) to both humans and common marmosets.^15^ Subsequent studies have confirmed the presence of meningeal lymphatic vessels in both human and animal models.^14^, ^16^, ^17^ Glymphatic activity is closely associated with sleep, aging, and cerebrovascular diseases^18^ and may represent a critical link in early AD pathogenesis. Additionally, Ding et al. observed impaired meningeal lymphatic vessel drainage function in Parkinson's disease in humans and mice.^16^

## 2 ANATOMY AND FUNCTIONAL ROLE OF THE GLYMPHATIC SYSTEM

The glymphatic system is a specialized lymphoid network that facilitates the dynamic circulation of the CSF and ISF and serves to eliminate metabolic waste and toxic proteins from the central nervous system.

### 2.1 Anatomical basis(Figure 1)

#### 2.1.1 Perivascular space

Perivascular spaces are fluid‐filled spaces in annular compartments formed between the outer layer of the cerebral arteries and the astrocytic endfeet; they serve as the primary conduit for CSF influx into the brain parenchyma.^19^ Their structural integrity is maintained by laminin and collagen within the basement membrane. High‐resolution two‐photon imaging has demonstrated rapid CSF flow along the perivascular spaces.^20^, ^21^ A recent racer study has demonstrated that macromolecules injected into the CSF migrate from periarterial to perivenous spaces at the arteriovenous overlapping zones within the leptomeninges.^22^ These perivascular pathways remain functional in amyloidosis mouse models and play a critical role in CSF clearance.^22^

#### 2.1.2 Astrocytic endfeet

Astrocytic endfeet form a continuous membranous sheath enveloping cerebral microvessels. These endfeet exhibit abundant AQP4 expression, whose polarized plasma membrane distribution localizing to perivascular astrocytic endfeet and epithelial basolateral membrane of cells drive ISF dynamics.^23^, ^24^ Notably, the perivascular AQP4 pool exhibits a 40‐fold higher density compared with the somal membrane, generating an osmotic sink effect. AQP4 thus mediates osmotically driven water flux from periarterial spaces into the interstitium, establishing a directional bulk flow between the cerebral microvasculature and brain parenchyma, critical for parenchymal clearance.^25^ This structural specialization is governed by the dystrophin‐associated protein complex (DAPC), which anchors AQP4 to the endfoot membrane via α‐syntrophin.^26^ Recent optogenetic studies reveal that pulsatile AQP4‐mediated water flux synchronizes with cerebral arterial vasomotion, suggesting a hemodynamic coupling mechanism.^27^ Aqp4^−/−^ mice exhibit an approximately 70% reduction in interstitial solute clearance.^28^ In the aging brain, AQP4 is delocalized from astrocyte endfeet, a phenomenon also implicated in glymphatic dysfunction.^29^ Furthermore, β‐amyloid accumulation has been shown to induce AQP4 delocalization.^30^

#### 2.1.3 Perivenous drainage and meningeal lymphatics

Metabolite‐laden ISF drains from the brain parenchyma via perivenous spaces into meningeal lymphatic vessels (e.g., dural lymphatic vasculature). The nasopharyngeal lymphatic plexus (NPLP) also serves as a critical pathway for CSF drainage.^31^ Lymphatic vessels near the pituitary gland and cavernous sinus, the anterolateral dura mater adjacent to the middle meningeal artery and petrosquamous sinus, and those around the cribriform plate can initially drain into the NPLP before ultimately reaching the dcLNs.^31^

### 2.2 Functional mechanism: Three‐phase model of CSF‐ISF circulatory pathway

#### 2.2.1 CSF influx

CSF enters the brain parenchyma via periarterial spaces, driven by two complementary hydrodynamic forces: (1) arterial pulsation from cardiac systolic pressure waves^32^, ^33^ and (2) respiratory oscillations transmitting intrathoracic pressure gradients to the cranial cavity.^34^, ^35^, ^36^ Notably, nonrapid eye movement (NREM) sleep enhances this CSF influx^37^, ^38^, ^39^ through a 20% increase in cerebral blood flow, which dilates penetrating arterioles and amplifies pulsatile energy transfer, as well as synchronized neuronal slow‐wave activity, which potentiates neurovascular coupling efficiency.

#### 2.2.2 ISF mixing and waste clearance

Following CSF‐ISF mixing in the brain parenchyma, solute transport is primarily mediated by AQP4 localized at astrocytic endfeet.^23^, ^40^ This mechanism enables the efficient clearance of macromolecular metabolites, including Aβ, Tau proteins, and lactic acid, into perivenous lymphatic compartments. Studies have demonstrated that CSF enters the brain parenchyma via the paravascular space surrounding the perforating arteries, and that ISF is cleared through the paravenous drainage pathways.^28^, ^41^ In AQP4‐deficient mice, a significant reduction in tracer movement into the brain parenchyma was observed compared with wild‐type (WT) controls at 30 min after intracisternal injection.^28^ Furthermore, mice deficient in AQP4 specifically in astrocytes exhibited a significant CSF inflow reduction and a 70% decrease in interstitial solute clearance.^28^

#### 2.2.3 Perivenous and lymphatic drainage pathways

Waste‐laden ISF drains from the brain parenchyma via perivenous spaces into meningeal lymphatic vessels, ultimately being cleared through cervical lymph nodes and systemic circulation.^13^, ^41^ Meningeal lymphatic vessels have been identified and confirmed in both rodents and humans.^12^, ^15^ Louveau et al.^12^ demonstrated that Evans Blue administered via intracerebroventricular injection in adult mice filled vessels adjacent to the internal jugular vein, entering the dcLNs within 30 min. Da Mesquita et al.^42^ demonstrated that ablating meningeal lymphatics in mice, followed by intracisternal injection of fluorescently labeled ovalbumin (OVA‐A647, ∼45 kDa) into the cerebellomedullary cistern, resulted in significantly reduced tracer drainage from the CSF to dCLNs, underscoring the functional role of these vessels in CSF‐immune system communication. Importantly, the major lymphatic structures of the dura mater have been identified in the human body, with direct connections observed between the lymphatic pathways along cranial nerves and vascular structures and the cervical lymph nodes.^14^ Additionally, the olfactory‐cribriform plate (or nasopharyngeal lymphatic plexus) may serve as another critical pathway for cerebrospinal fluid or lymphatic drainage^31^(Figure 2). Since CSF can eventually drain into the dcLNs or superficial cervical lymph nodes (scLNs) via meningeal lymphatic vessels, the driving force behind its drainage remains poorly understood. Conversely, whether the peripheral lymphatic network influences CSF homeostasis is also unclear. The current review focused on where meningeal lymphatic vessels drain. Papadopoulos et al.^43^ demonstrated that CSF outflow to dcLNs is spontaneous and closely related to intracranial pressure, whereas its drainage to scLNs is pump‐driven. Impaired drainage to the dcLNs can disrupt CSF homeostasis, leading to obstructed CSF outflow.

**FIGURE 1:**
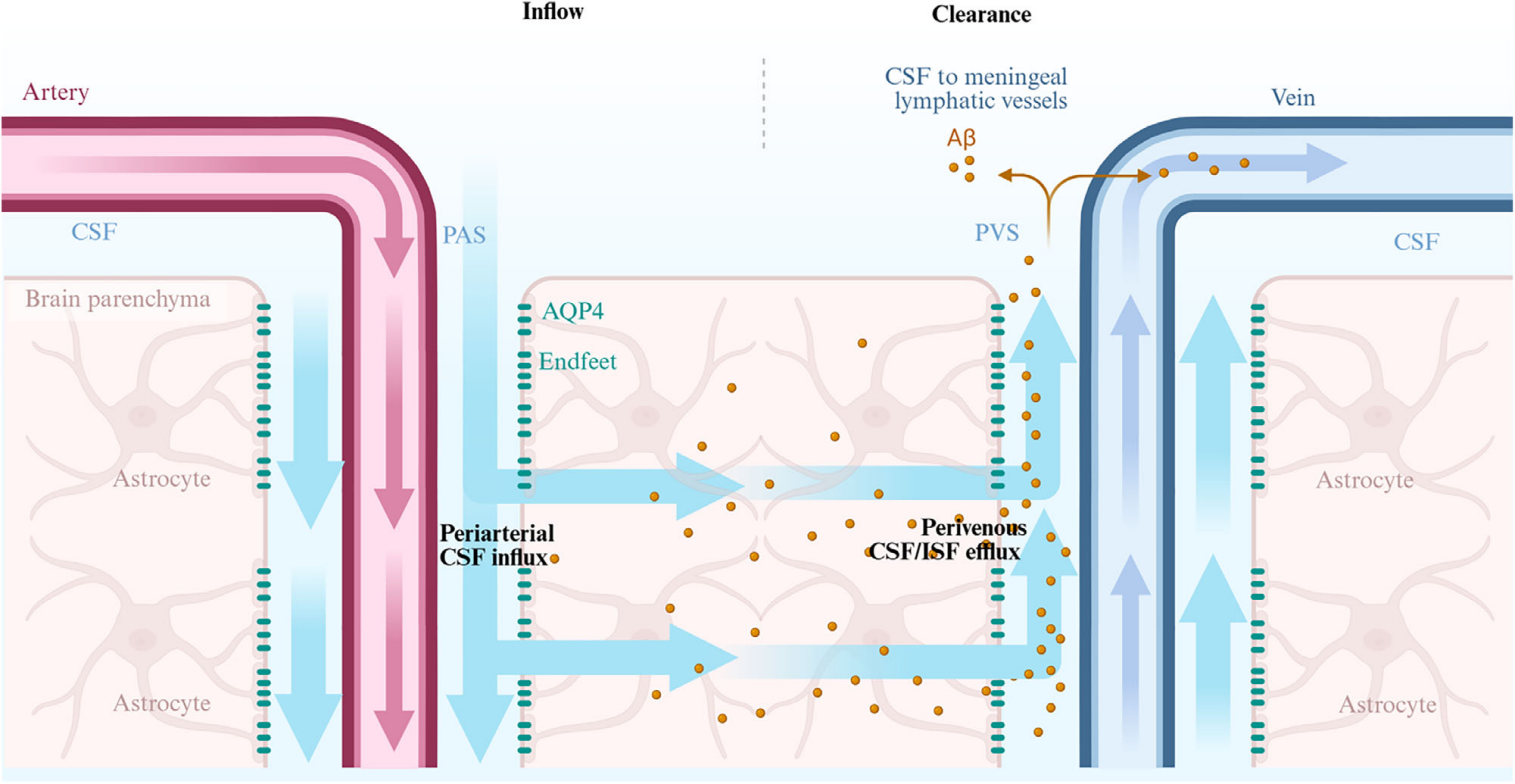
Composition and mechanism of the glymphatic system. The glymphatic system is a specialized waste clearance pathway in the central nervous system, comprising three key anatomical components: (A) Periarterial space. (B) Astrocytic networks in brain parenchyma. (C) Perivenous space. CSF enters the brain parenchyma via the arterial perivascular space through AQP4 expressed on astrocytic endfeet. Within the parenchymal interstitium, CSF mixes with ISF, enabling metabolic exchange and waste product clearance. The CSF‐ISF mixture subsequently drains through the venous perivascular space, ultimately flowing to the meningeal lymphatic vessels for systemic circulation and clearance. Diagram created using BioRender. AQP4, aquaporin‐4; CSF, cerebrospinal fluid; ISF, interstitial fluid; PAS, periarterial space; PVS, perivenous space.

**FIGURE 2:**
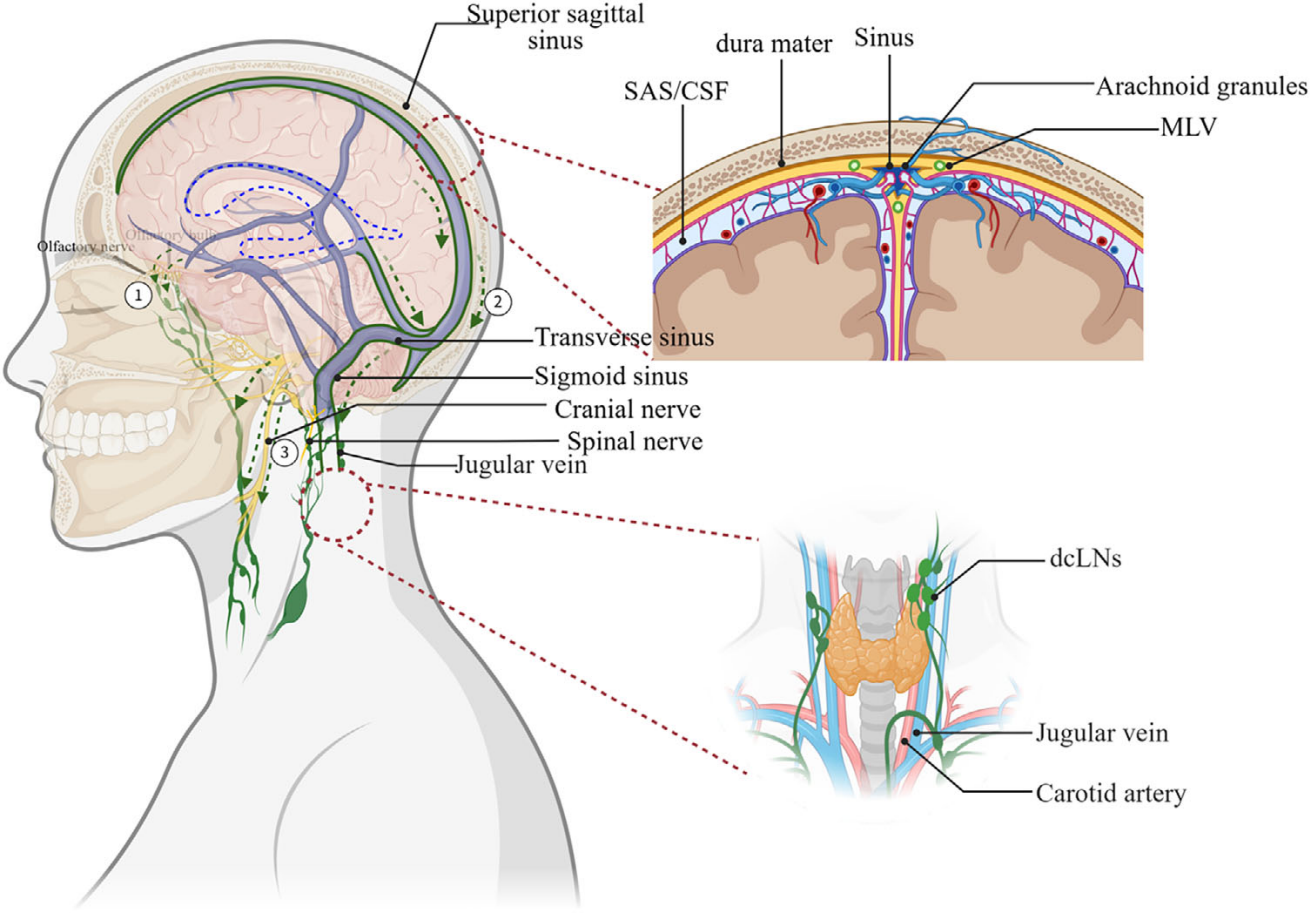
Classical glymphatic drainage pathways. (A) Olfactory–cribriform plate pathway for intracranial lymphatic drainage: Cribriform plate along olfactory nerves → nasal → lymphatic vessels → retropharyngeal lymph nodes → superficial/deep cervical lymph nodes (drainage terminus). (B) Meningeal lymphatic–venous pathway: Meningeal lymphatic vessels accompanying venous sinuses/dural veins (direct drainage) → deep cervical lymph nodes. (C) Perineural pathway for lymphatic drainage: Perivascular spaces of spinal or cranial nerves → regional lymph nodes (e.g., cervical lymph nodes). Diagram created using BioRender. CSF, cerebrospinal fluid; dcLNs, deep cervical lymph nodes; MLV, meningeal lymphatic vessels; SAS, subarachnoid space.

**FIGURE 3:**
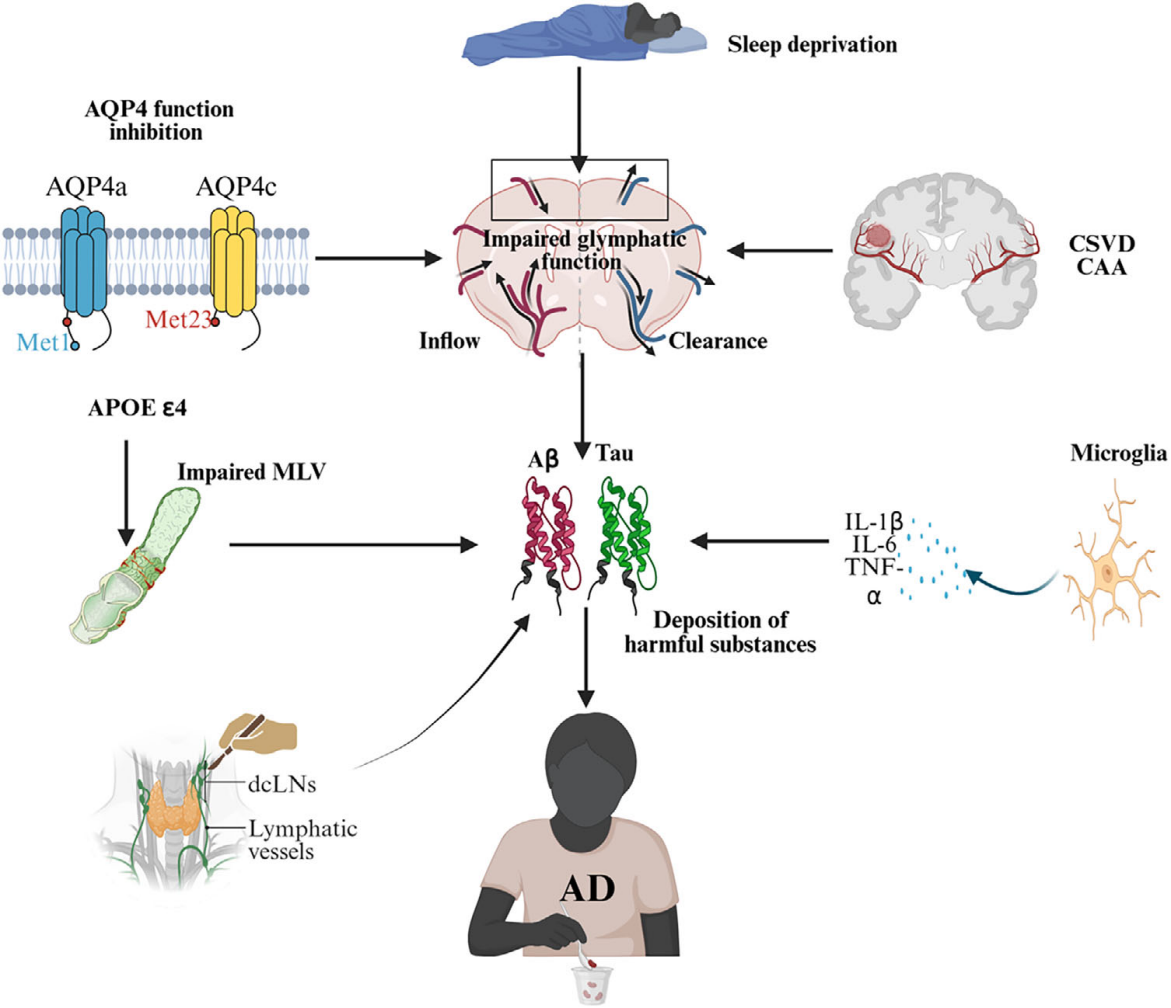
Schematic of mechanistic link between impaired glymphatic–meningeal lymphatic function and the pathogenesis of Alzheimer's disease. Key pathophysiological interactions: Glymphatic dysfunction resulting from AQP4 channel impairment/mislocalization, cerebral small vessel disease, sleep architecture disruption, meningeal lymphatic impairment associated with the APOE ε4 genotype, iatrogenic disruption through cervical lymph node dissection, and neuroinflammatory‐mediated clearance inhibition. Collectively, these impairments lead to the pathological accumulation of neurotoxic metabolites (e.g., amyloid‐β, tau proteins), creating a vicious cycle that exacerbates Alzheimer's disease progression. Diagram created using BioRender. APOE, apolipoprotein E; AQP4, aquaporin‐4; Aβ, amyloid beta; CAA, cerebral amyloid angiopathy; CSVD, cerebral small‐vessel disease; dcLNs, deep cervical lymph nodes; MLV, meningeal lymphatic vessels.

### 2.3 Core molecular mechanism: Polarized localization and regulation of AQP4

AQP4 serves as a central molecular hub for the glymphatic system. Its polarized distribution,^44^ characterized by its dense clustering at the astrocytic endfeet, establishes a unidirectional osmotically driven water flux. This spatial polarization creates a hydrostatic pressure differential that facilitates convective solute clearance through perivascular pathways. The polarized distribution of AQP4 at astrocytic endfoot membranes is orchestrated by a multitiered regulatory framework:

(1) Anchoring: AQP4 is anchored at endfeet via α‐syntrophin‐dependent targeting and the DAPC via PDZ domain interactions.^23^ (2) Cytoskeletal coupling: It maintains AQP4 stability via the F‐actin cytoskeleton. Notably, F‐actin depolymerization accompanied by connexin 43 (Cx43) downregulation was detected in AQP4‐knockdown mice.^45^ (3) Transcriptional regulation: Wnt/β‐Catenin Signaling; The study revealed an upregulation of AQP4 expression in β‐catenin knockout mice.^46^ (4) Inflammatory cytokines: Tumor necrosis factor (TNF) ‐α activates the NF‐κB pathway, promotes the binding of p65 to the AQP4 gene promoter region, enhances AQP4 expression, and ultimately reduces the viability of astrocytes.^47^ (5) Posttranslational modifications. Phosphorylation: PKC and dopamine‐mediated Ser180 phosphorylation reduce the water permeability.^48^ (6) Dystrophin 71 (DP71): Its deficiency critically disrupts AQP4 polarization and compromises lymphatic function.^49^ Mice with selective DP71 deletion exhibited a dramatic 70% reduction in the polarized distribution of AQP4 channels.^50^ (7) In addition, astrocytes additionally sustain the intracellular and extracellular K+ gradient via the Kir4.1 potassium channel, which, in conjunction with AQP4, regulates osmotic pressure and facilitates ISF flow.^51^, ^52^ (8) Inhibition of the platelet‐derived growth factor‐B (PDGF‐B) signaling pathway attenuates AQP4 polarity formation and impairs glymphatic system maturation.^53^

## 3 DYNAMIC REGULATORY FACTORS OF THE GLYMPHATIC SYSTEM

### 3.1 Circadian rhythms and sleep

Sleep disorders can affect AD risk and vice versa. Sleep has been shown to increase the interstitial volume of cells, thereby significantly enhancing the CSF‐ISF convective exchange. This increased ISF flow facilitates the clearance of beta‐amyloid proteins during sleep.^54^, ^55^

### 3.2 Vascular dynamics

Arterial pulsations‐mechanical waves generated by cardiac cycles‐serve as the primary driver of CSF flow.^56^ Iliff et al.^20^ demonstrated that cerebral arterial pulsatility drives paravascular CSF‐ISF exchange. They used two‐photon microscopy was to visualize arterial wall pulsatility along the penetrating intracortical arteries. They then demonstrated that internal carotid artery ligation slowed paravascular CSF‐ISF exchange rates, whereas dobutamine administration accelerated this process. These results provided direct experimental evidence establishing cerebral arterial pulsatility as a critical driver of CSF influx through the paravascular pathway and its subsequent parenchymal distribution. All these suggest that changes in arterial pulsation may contribute to toxic solute accumulation and deposition.^20^, ^56^, ^57^ Arteriolosclerosis impairs glymphatic function in animal models, with enlarged perivascular spaces as a potential morphological manifestation of system dysregulation, with enlarged perivascular spaces a likely structural biomarker of impaired glymphatic clearance.^58^ The comorbidity of arteriosclerosis and hypertension is recognized as a significant risk factor for AD.^59^ This association may be mechanistically linked to arteriosclerosis‐induced attenuation of vascular pulsatility, which impairs CSF–ISF exchange. Such dysfunction likely contributes to insufficient clearance of neurotoxic waste products (e.g., Aβ), thereby exacerbating the pathological hallmarks of AD.

### 3.3 External interventions

Body positioning affects glymphatic function: The lateral decubitus position (particularly right sided) optimizes CSF flow pathways. In murine models, Aβ clearance efficiency is 25% higher in lateral versus supine positioning.^60^ Aerobic exercise enhances waste clearance via increased cerebral blood flow and lymphatic contraction frequency. Clinical studies report a 15% increase in CSF flow velocity in older adults performing 150 min/week of moderate‐intensity exercise.^61^

## 4 THE GLYMPHATIC SYSTEM AND AD (FIGURE 3)

The glymphatic system, a brain‐wide waste clearance network mediated by astrocyte‐dependent CSF‐ISF exchange, is critically implicated in AD pathogenesis.^62^, ^63^, ^64^, ^65^, ^66^ Its dysregulation causes the accumulation of neurotoxic proteins (e.g., Aβ and tau) and exacerbates neurodegenerative cascades.

### 4.1 Aβ clearance obstruction: Central role of the glymphatic system

Abnormal Aβ accumulation in the brain is a key pathological feature of AD pathogenesis. Bateman et al.^67^ first reported that Aβ dissociation production and clearance in the human central nervous system were 7.6%/h and 8.2%/h, respectively. The amyloid hypothesis posits that an imbalance between Aβ production and clearance represents the main causative factor underlying AD development.^68^, ^69^ Glymphatic influx facilitates soluble Aβ removal from the brain parenchyma into meningeal lymphatic vessels. Both decreased AQP4 expression and disrupted polarity have been shown to diminish glymphatic system functionality.^53^ In animal models, glymphatic suppression (via AQP4 knockout or sleep deprivation) elevates Aβ plaque deposition.^28^, ^54^ AQP4^−^/^−^ mice demonstrated a 50% decrease in Aβ clearance efficiency compared with WT controls, with amyloid plaque deposition significantly accelerated. Furthermore, the retention time of Aβ42 in the brain parenchyma of AQP4^−^/^−^ mice was prolonged to 2‐3 fold that of WT mice.^28^ In WTC57BL/6J mice treated with the AQP4 inhibitor TGN‐020, perivascular Aβ40 accumulation significantly increased.^70^ Dynamic contrast‐enhanced MRI (DCE‐MRI) revealed a negative correlation between the glymphatic influx rate of CSF and CSF Aβ42 levels in patients with AD, along with a significant inverse association with Aβ‐positron emission tomography (PET) signal intensity.^71^ *Post mortem* studies have demonstrated that the polarized distribution of AQP4 (endfeet/soma expression ratio) in patients with AD was 40% lower than in healthy controls and negatively correlated with Braak staging (neuropathological severity).^72^

In a 20‐year longitudinal study of patients with sporadic AD (SAD), Jianping et al. demonstrated that compared with cognitively normal controls, individuals who later developed AD exhibited significantly altered CSF Aβ levels as early as 18 years prior to clinical diagnosis, with abnormal Aβ42/40 ratio changes detectable 14 years before diagnosis.^73^ Furthermore, CSF biomarker dynamics in the AD cohort displayed a biphasic pattern: accelerated pathological changes during the early preclinical stage, followed by progressive deceleration as cognitive impairment advanced.^73^

### 4.2 Tau protein accumulation in brain regions

Tau is a 441‐amino acid microtubule‐associated protein rich in proline and serine residues, which provide abundant phosphorylation sites.^74^ Its primary function is to stabilize microtubules, promote tubulin polymerization, and prevent microtubule depolymerization, thereby maintaining axonal structural integrity. Additionally, tau protein regulates axonal transport through kinase‐phosphatase‐mediated phosphorylation balance. This regulation modulates its binding affinity to microtubules, consequently altering microtubule‐dependent intracellular transport. Tau hyperphosphorylation can significantly reduce its affinity for microtubules, leading to microtubule destabilization caused by conformational changes and misfolding of tau's normal structure,^74^, ^75^ ultimately triggering the formation of neurofibrillary tangles, a hallmark of AD pathology.^76^, ^77^

Glymphatic stagnation promotes extracellular aggregation of hyperphosphorylated tau, enabling its transsynaptic spread. MRI studies in humans reveal enlarged perivascular spaces perivascular spaces, a marker of glymphatic impairment, correlating with tau burden in AD.^71^ A recent study revealed that plasma p‐tau231 and p‐tau217 can be detected prior to Aβ plaque pathology, suggesting their utility as blood‐based biomarkers for preclinical populations in AD.^78^ Barthélemy et al. demonstrated that plasma %p‐tau217 (i.e., the ratio of phosporylated‐tau217 to nonphosphorylated tau) exhibits clinical performance comparable to or superior to FDA‐approved CSF tests currently used in clinical practice for detecting AD pathology.^79^

Impaired glymphatic system function may accelerate the pathological tau accumulation.^71^, ^80^ Intraperitoneal administration of TGN‐020 (an AQP4 inhibitor, 250 mg/kg) in mice, when preceded by intrastriatal infusion of tau‐containing brain homogenate 15 min prior, resulted in glymphatic CSF‐ISF exchange dysfunction and reduced pathogenic tau clearance, ultimately leading to its pathological accumulation.^71^ Lopes et al. inoculated tau proteins into the hippocampus and cortex of P301S mice, followed by treatment with 50 mg/kg TGN‐020 (3 doses/week, intragastric) for 10 weeks. They discovered that after impairment of lymphatic drainage inhibition, tau aggregation in the brain increased, and the mice exhibited significantly impaired recognition memory.^80^

### 4.3 The vicious cycle of neuroinflammation

There is a complex interplay between inflammation and AD. Neuroinflammation is increasingly recognized as not merely a consequence of AD pathology but may also drive AD progression. Microglia, the resident immune cells of the brain, play a pivotal role in central nervous system immunity. Microglial dysfunction is closely associated with neurodegenerative disorders such as AD and Parkinson's disease.^81^, ^82^ In AD, brain‐resident immune cells, particularly microglia, undergo sustained activation and release proinflammatory cytokines (e.g., interleukin [IL] ‐1β, IL‐6, and TNF‐α) and reactive oxygen species. Elevated TNF‐α and IL‐1β levels are closely associated with cholinergic dysfunction and cognitive decline in AD.^83^ In patients with AD, elevated levels of inflammatory cytokines IL‐1β, IL‐6, and TNF‐α have been observed, which are associated with abnormal Aβ accumulation.^84^, ^85^, ^86^ This further perpetuates proinflammatory responses, ultimately resulting in a vicious cycle of neuroinflammation and amyloid pathology progression.^84^

### 4.4 dcLN dissection increases AD risk

The dcLNs clear metabolic waste products and neurotoxic substances from brain ISF and CSF.^13^, ^14^, ^42^ In the APP/PS1 mouse model of AD, Wang et al.^87^ demonstrated that ligation of the deep cervical lymphatics exacerbated cognitive deficits, accompanied by elevated cerebral Aβ accumulation, neuroinflammation, synaptic protein loss, and impaired AQP4 polarization. A recent study revealed mild cognitive impairment accompanied by significant anxiety‐ and depression‐like behaviors among C57BL/6 mice undergoing deep cervical lymph node dissection; moreover, cervical lymph node dissection accelerated p‐tau deposition in young adult mice.^88^ Chao et al.^89^ identified nine cases of postoperative dementia of 251 patients with head and neck cancer undergoing cervical lymph node dissection (mean postoperative follow‐up period: 50.1 ± 35.3 months). These findings suggest that surgical disruption of the dcLNs may elevate dementia/AD risk. In contrast, Xie et al.^90^ and Li et al.^91^ demonstrated that deep cervical lymphaticovenous anastomosis (LVA) could ameliorate dementia symptoms in patients with AD through enhanced cerebral lymphatic drainage.

### 4.5 Cross effects of vascular dysfunction and the glymphatic system

AD is frequently comorbid with cerebral amyloid angiopathy (CAA)^92^ and vascular sclerosis,^93^ both of which synergistically impair glymphatic function. Impairment of the glymphatic system and clearance dysfunction have also been observed in dementia associated with cerebral small‐vessel disease (CSVD).^94^ Aβ deposition in vascular walls compresses periarterial spaces, reducing CSF influx. Charidimou et al.^95^ demonstrated that perivascular spaces in patients with CAA and hypertensive arteriopathy was assessed using MRI. Their findings revealed that perivascular spaces in specific brain regions, such as the centrum semiovale, were significantly enlarged in patients with CAA. This enlargement may be associated with meningeal vascular pathology and impaired ISF drainage due to β‐amyloid deposition. Vestergaard recently identified a robust association between Aβ accumulation and impaired cerebrovascular function. These findings suggest that the cerebrovascular dysfunction induced by Aβ accumulation may represent an early pathological mechanism contributing to neurodegenerative diseases.^96^ In addition, astrocytic AQP4 plays a crucial role in maintaining CSF homeostasis and lymphatic clearance systems (Pathophysiology and probable etiology of CSVD in vascular dementia and AD.^97^ Mislocalization or altered expression of the AQP4 gene has been observed in patients with small‐vessel disease.^98^

In spontaneously hypertensive stroke‐prone rat models (SHRSP), the accumulation of Aβ and tau proteins was observed as the animals aged, indicating that hypertension‐associated small‐vessel disease may contribute to age‐related Aβ clearance impairment, elevated AβPP expression, and tau hyperphosphorylation in neurons.^99^ Similarly, Bueche et al. reported the accumulation of Aβ in the brains of SHRSP.^100^ Hypertension is established as a significant risk factor for AD, supported by extensive epidemiological and mechanistic evidence.^93^, ^101^, ^102^, ^103^ Furthermore, DCE‐MRI reveals an 18% reduction in CSF flow velocity in hypertensive individuals,^104^ suggesting that vascular‐lymphatic coupling dysfunction may underlie this association.

### 4.6 Regulatory role of apolipoprotein E genotypes

The apolipoprotein E (APOE) 4 allele, the strongest genetic risk factor for AD, is mechanistically linked to glymphatic dysfunction.^105^, ^106^, ^107^ In fact, research has demonstrated that the APOE gene is highly expressed in meningeal lymphoendothelial cells.^108^ Carrying the APOE epsilon 4 allele has been associated with a 2‐ to 12‐fold increased AD risk.^109^, ^110^, ^111^ Mentis et al. proposed that the APOE **ε**4 allele may contribute to functional and structural abnormalities in meningeal lymphatic vessels. This impairment could subsequently result in diminished Aβ clearance, other macromolecules, inflammatory mediators, and immune cells from the brain, ultimately exacerbating the progression of AD manifestations.^106^ Mouse experiments have revealed that APOE4 directly impairs the blood‐brain barrier, reducing Aβ clearance, thereby suggesting that APOE‐related neurovascular dysfunction may also contribute to AD.^112^

### 4.7 Lymphatic mechanisms linking sleep disturbances to AD risk

Sleep deprivation is an independent risk factor for AD, with its pathological mechanisms strongly associated with suppressed glymphatic function.^113^, ^114^ Sleep represents an innate physiological protective mechanism, with glymphatic clearance exhibiting time‐dependent and cyclical regulation. During NREM sleep, the CSF transport is initiated and maintained.^54^ Compared with the wakeful state, the sleeping brain demonstrates enhanced efficiency in eliminating metabolic waste products accumulated during periods of active wakefulness.^114^ Individuals with sleep disorders are at an increased risk of neurodegenerative diseases, such as AD.^113^, ^115^ Sleep deprivation in mice significantly suppressed CSF influx into the brain and its subsequent clearance from the interstitial space.^116^ Sleep deprivation has been associated with CSF Aβ level fluctuations and its deposition in the brain.^117^ Notably, even a single night of sleep deprivation reduced CSF Aβ42 levels by 6%.^118^ (Elevated CSF Aβ42 levels show independent association with attenuated cognitive decline.^119^)

## 5 THERAPEUTIC STRATEGIES FOR REGULATING THE BRAIN GLYMPHATIC SYSTEM

Excessive Aβ deposition is a critical pathological hallmark of AD. The development of Aβ‐targeted disease‐modifying therapies has long been a major focus for researchers and clinicians, with the premise that Aβ reduction could ameliorate cognitive impairment in AD. Currently, three anti‐Aβ monoclonal antibodies‐lecanemab, aducanumab, and donanemab‐have received approval for AD treatment, demonstrating the potential to alleviate symptoms and slow disease progression in mild patients with AD.^120^ However, these therapies are associated with significant adverse effects,^8^ including cerebral edema/hemorrhage risks, infusion‐related reactions, and accelerated brain atrophy, alongside limited efficacy. Recent reports have indicated that lecanemab shows measurable clinical benefits in only 27% of patients with mild AD.^8^ In summary, whereas Aβ‐targeted disease‐modifying therapies exhibit modest therapeutic effects, their substantial safety concerns persist‐a scenario akin to firefighters extinguishing a fire only to have the house collapse in the process. The critical challenge remains identifying therapeutic strategies that achieve robust cognitive improvement without compromising safety. Emerging evidence suggests that glymphatic system‐targeted interventions that enhance cerebral waste clearance may represent a promising frontier for future AD therapeutics.

### 5.1 Targeted therapy for precise regulation of glymphatic function

#### 5.1.1 Enhance AQP4 function and promote CSF and ISF flow

Design specific interventions targeting key molecules of the glymphatic system (such as AQP4 and lymphatic vessel markers) and regulatory pathways. Dong et al.^40^ discovered impaired glymphatic system function in a mouse model of perioperative neurocognitive disorders. Application of adeno‐associated virus to overexpress AQP4 enhanced AQP4 polarization, which augmented lymphatic function and ameliorated cognitive deficits. AQP4ex, one of the AQP4 isoforms, was investigated in AQP4ex‐KO mice through fluorescent Aβmicroinjection into brain parenchyma. Compared with the WT controls, Aβ clearance was significantly impaired in knockout mice, accompanied by prolonged dissemination distance to cervical lymph nodes and reduced fluorescence intensity, indicating deficient parenchymal drainage efficiency. The findings demonstrate that disruption of AQP4 polarization forces solute clearance to rely on slower passive diffusion mechanisms, substantially diminishing Aβ removal efficiency. The authors emphasize that enhancing the AQP4ex function may hold therapeutic implications for cerebral edema and neurodegenerative disorders such as AD.^121^

#### 5.1.2 Enhance the function of meningeal lymphatic vessels

In 5xFAD mice (a model of AD), researchers observed progressive dysfunction of meningeal lymphatic vessels accompanied by increased Aβ deposition along these vessels. Using Visudyne‐mediated photodynamic therapy (Vis/photo) to establish a meningeal lymphatic vessel ablation model, the study demonstrated that lymphatic ablation (Vis/photo) accelerated Aβ deposition whereas significantly impairing cognitive and memory functions. Furthermore, intracerebroventricular administration of mAdu revealed markedly reduced binding between mAdu and Aβ in brain parenchyma of mice with ablated meningeal lymphatics. Notably, combined treatment with murine vascular endothelial growth factor‐C (VEGF‐C) to enhance meningeal lymphatic function and aducanumab administration synergistically reversed both amyloid pathology and cognitive deficits in the AD model mice.^122^

### 5.2 Promote glymphatic flow and waste clearance‐deep cervical LVA

On the basis of the brain‐peripheral lymphatic interaction theory, Chinese researchers pioneered LVA, a groundbreaking microsurgical technique that anastomoses deep cervical lymphatic vessels with adjacent veins to reconstruct drainage pathways for cerebral metabolic waste.^90^, ^91^ Developed by Xie Q. and colleagues,^90^ LVA led to significant behavioral and cognitive memory improvements at the 9‐month postoperative follow‐up in 50 patients. In a subsequent study, researchers from the Shanghai Mental Health Center, China, applied cervical shunting to unclog cerebral lymphatic systems (CSULS) in a 70‐year‐old patient with AD.^91^ At 5 weeks postoperatively, cognitive assessments revealed significant enhancements: Mini‐Mental State Examination (MMSE) scores increased from 5 to 7 points, Clinical Dementia Rating Sum of Boxes (CDR‐SB) improved from 10 to 8, and the Geriatric Depression Scale score decreased from 9 to 0. Additionally, sleep quality enhanced and daytime alertness improved postoperatively, suggesting that improved lymphatic drainage may indirectly regulate neural rhythmic activity. A recent clinical trial involving 26 cases concluded that approximately 60% of caregivers observed varying degrees of symptom improvement in patients 1 month after LVA surgery. Postoperative MMSE scores of the patients were significantly higher than preoperative scores, whereas 15% of the patients exhibited increased cognitive assessment (Montreal Cognitive Assessment [MoCA]) scores, and 42% exhibited a decrease in Neuropsychiatric Inventory (NPI)scores.^123^ In China, LVA has rapidly expanded, with implementation in over 200 medical institutions across departments, including microsurgery, plastic surgery, and neurosurgery.^124^

### 5.3 Targeting the glymphatic system as an early intervention strategy

In the preclinical stage of AD (e.g., Aβ‐positive asymptomatic individuals), modulating glymphatic function may delay pathological progression. High‐risk populations with glymphatic hypofunction can be identified using dynamic MRI or CSF hydrodynamic metrics, enabling the timely initiation of early interventions. Furthermore, artificial intelligence (AI)‐driven predictive models integrating multiomics data (genomic, imaging, and metabolomic profiles) could be leveraged to construct a glymphatic dysfunction risk stratification score for AD. This approach would guide personalized preventive therapies, such as lifestyle modifications, pharmacological enhancers of glymphatic clearance, or noninvasive neuromodulation, to restore cerebral waste drainage before irreversible neurodegeneration occurs. Such strategies represent a shift from symptomatic management to preemptive disease modification in AD.

### 5.4 Optimization and standardization of nonpharmacological interventions

Under the guidance of glymphatic system physiological regulation principles, lifestyle‐based intervention strategies are proposed to enhance cerebral waste clearance and decelerate AD progression. (1) Sleep‐wake cycle engineering: A closed‐loop transcranial electrical stimulation (tES) system can be used to precisely augment slow‐wave sleep (SWS)^125^‐a critical phase for lymphatic activation. Wearable devices enabling real‐time monitoring of lymphatic function biomarkers (e.g., heart rate variability and CSF pulsatility frequency) may facilitate adaptive neuromodulation targeting individualized sleep architecture. (2) Postural and exercise modulation: Evidence‐based “glymphatic‐optimized postures” (e.g., lateral decubitus positioning during sleep)^60^ and personalized exercise regimens^126^, ^127^ (e.g., intermittent aerobic training, tai chi, and yoga) should be standardized. (3) Mechanical stimulation of the superficial cervical lymph nodes has been demonstrated to enhance CSF drainage.^128^ This noninvasive technique may play a significant role in AD prevention and treatment. For instance, bilateral cervical massage could potentially benefit patients with AD by improving CSF clearance and reducing pathological protein accumulation. (4) Photostimulation may offer a potential therapeutic direction for AD. In 5xFAD mouse models, 40 Hz blue light was shown to prevent memory decline and motivation loss, possibly by activating the vLGN/IGL‐Re visual pathway and restoration of aquaporin polarity. Future light‐based therapies may be promising for AD treatment.^64^

Future intelligent algorithm‐based multimodal physiological feedback systems incorporating MRI‐derived CSF hemodynamics and nocturnal cerebral oxygenation patterns, thereby enabling presymptomatic state assessment in patients with AD.

### 5.5 Personalized and population‐specific therapeutic strategies

Therapeutic regimens should be customized based on AD subtypes, genetic profiles, and comorbid conditions. AD risk is closely associated with the APOE gene. APOE has three alleles: ε2, ε3, and ε4. Bello‐Corral et al.^129^ reported a higher ApoE3 (74%) prevalence in patients with AD, followed by ApoE4 (22%). APOE2 appears to function as a protective genetic variant against AD pathogenesis.^130^ Patients with AD carrying the APOE4 allele have an increased risk of ARIA‐E or ARIA‐H when treated with lecanemab.^131^ Future therapeutic development and preclinical evaluation for AD should rigorously incorporate APOE allele profiling to assess intervention‐specific risk–benefit ratios, thereby minimizing iatrogenic exacerbation of neuropathological trajectories. Genotype‐stratified precision therapeutics targeting APOE‐related molecular cascades may constitute a pivotal paradigm for next‐generation disease‐modifying strategies. In addition, for patients with AD with comorbid hypertension or diabetes, a combined regimen of antihypertensives (e.g., angiotensin receptor–neprilysin inhibitors [ARNI]) and lymph‐activating agents is proposed to synergistically improve vascular elasticity and ISF dynamics.

## 6 DEVELOPMENT OF NONINVASIVE GLYMPHATIC MONITORING TECHNOLOGIES

Preclinical assessment of AD is critical because early screening may enable timely interventions, thereby improving patients' overall prognosis. Recently, Chu et al.^132^ pioneered the use of the Florey Dementia Index (FDI), a noninvasive assessment tool, to predict the age of onset for both mild cognitive impairment and AD. Advancements in noninvasive techniques for assessing human lymphatic function hold transformative potential for the preclinical diagnosis of neurodegenerative diseases. These innovations, including diffusion tensor imaging along the perivascular space (DTI‐ALPS), perivascular space analysis, and free water index, have significantly enhanced our ability to investigate glymphatic system functionality and its alterations in pathological conditions.^133^, ^134^, ^135^ The DTI‐ALPS index, derived from MR diffusion tensor imaging, enables quantitative analysis and assessment of glymphatic system functional activity.^134^ This imaging biomarker shows potential for radiologically evaluating and detecting patients with early‐stage AD through noninvasive characterization of perivascular clearance dysfunction associated with disease pathogenesis. Noninvasive methods based on MRI and quantification of AQP4 in CSF may also be valuable for evaluating glymphatic system functionality.^135^

Future emerging modalities, such as ultrafast ultrasound CSF flow imaging,^136^ dynamic optical coherence tomography,^137^ and wearable magnetoencephalography systems,^138^ could enable continuous, quantitative assessment of glymphatic efficiency. These tools may enable the detection of early impairments in clearance mechanisms prior to amyloid plaque deposition. When integrated with AI‐driven hydrodynamic modeling, such approaches can generate predictive biomarkers for presymptomatic AD interventions, addressing a critical gap in current preventive neurology frameworks.

## 7 CONCLUSION

The glymphatic system is a paradigm shift in AD therapeutics, transitioning from “clearance of pathological proteins” to “system‐level modulation.” Breakthroughs in in vivo imaging and AI predictive models can establish multidimensional interventions targeting the brain's glymphatic network for precision medicine in AD. Achieving this goal requires a deep integration of neuroscience, engineering, and clinical medicine, coupled with sustained collaboration among interdisciplinary teams.

## AUTHOR CONTRIBUTIONS

All authors provided critical input for this review. All authors have reviewed the final version and agreed with the decision to submit.

## CONFLICT OF INTEREST STATEMENT

The authors declare no conflicts of interest. Author disclosures are available in the supporting information.

## Supporting information



Supporting Information
